# Impact of varicella vaccination on the epidemiology of herpes zoster and postherpetic neuralgia: a narrative review

**DOI:** 10.12701/jyms.2026.43.22

**Published:** 2026-03-18

**Authors:** Hyeon-Soo Park, Gyeong-Jo Byeon

**Affiliations:** 1 Department of Anesthesia and Pain Medicine, Pusan National University Yangsan Hospital, Pusan National University School of Medicine, Yangsan, Korea; 2 Research Institute for Convergence of Biomedical Science and Technology, Pusan National University Yangsan Hospital, Yangsan, Korea

**Keywords:** Herpes zoster, Immunity, Neuralgia, Vaccination, Varicella

## Abstract

Varicella-zoster virus (VZV) establishes lifelong latency following primary infection and can reactivate later in life to cause herpes zoster (HZ) and its debilitating complication postherpetic neuralgia (PHN). The implementation of varicella vaccinations has profoundly altered the epidemiological landscape of VZV. This narrative review examines the current literature to evaluate the impact of these vaccination programs on the incidence of HZ and PHN. Early mathematical models based on the “exogenous boosting” hypothesis predicted a substantial surge in adult HZ due to reduced exposure to circulating wild-type virus. However, long-term epidemiological data demonstrate that the incidence of adult HZ was already increasing prior to the introduction of vaccination programs and did not accelerate post-implementation, suggesting other primary drivers, such as an aging population. This review highlights the significant decline in HZ incidence among vaccinated pediatric populations, as the attenuated vOka vaccine strain is substantially less prone to reactivation than the wild-type virus. Furthermore, HZ that does occur in vaccinated individuals tends to be milder, resulting in a reduced risk of progression to PHN. To address the persistent risk in older adults, a recombinant zoster vaccine is recommended as a highly effective secondary prevention strategy. Despite challenges, such as breakthrough infections and the need for long-term monitoring of vaccine-induced immunity, varicella vaccination remains a cornerstone of public health, offering broad protection across different ages. Further research is needed to fully understand the long-term impact of varicella vaccinations on the incidence of HZ and PHN and to identify additional risk factors for these conditions.

## Introduction

Varicella, commonly known as chickenpox, is caused by the varicella-zoster virus (VZV). Following primary infection, the virus remains latent in the sensory ganglia and can reactivate as herpes zoster (HZ), particularly in older adults and immune-compromised individuals [1,2]. Reactivation can lead to a painful vesicular rash that may progress to postherpetic neuralgia (PHN), a chronic and debilitating condition characterized by persistent neuropathic pain [3].

The introduction of the varicella vaccine has significantly improved public health, leading to a substantial decrease in the incidence of varicella infections [4]. However, the broad implications of varicella vaccination on the epidemiology of HZ and PHN remain an area of active research and debate. Use of varicella vaccines has prompted discussions regarding their potential impact on HZ incidence, given the theoretical possibility of altering natural immunity dynamics [5]. Early hypotheses suggested that reduced exposure to wild-type VZV would increase the risk of HZ in adults previously infected with varicella [6]. However, subsequent studies have yielded mixed results, with some indicating an increase in HZ incidence and others finding no significant change.

This comprehensive review aimed to evaluate the effects of varicella vaccination on the epidemiology of HZ and PHN, to synthesize the current evidence, and to suggest implications for future vaccine policies.

## Varicella virus

Human herpesviruses are large, enveloped particles, typically measuring 100 to 200 nm in diameter. At the core, they possess a double-stranded DNA genome encased within an icosahedral capsid. These viruses are distinguished by their genetic complexity and contain between 70 and 200 open reading frames, placing them among the most diverse human viruses. In addition, the viral genome encodes numerous noncoding RNAs that play critical regulatory roles. This vast genetic toolkit allows the viruses to establish highly sophisticated and multifaceted interactions with host organisms. These pathogens possess the capacity to manipulate both the immune response and the host cells they infect. This ability stems from the comprehensive genetic repertoire of herpesviruses, which includes a significant proportion of noncoding RNAs derived from the viral DNA, all of which contribute to a diverse array of regulatory actions and foster an intricate host relationship. The alpha subfamily (including VZV and herpes simplex virus types 1 and 2) primarily establishes long-term latency within neurons. However, their replication in epithelial tissues is crucial for efficient transmission via the skin or mucosa. In contrast, the beta and gamma subfamilies preferentially target various leukocyte subsets, although they are also capable of infecting epithelial cells. Reactivation of these viruses is often linked to a mild feeling of general discomfort (malaise) but does not necessarily result in noticeable clinical symptoms (Table 1) [7].

VZV, otherwise designated human herpesvirus 3, is a highly contagious virus responsible for causing varicella, a disease with a high incidence in children worldwide, and HZ, a disease that affects adults [8]. VZV transmission occurs via several routes, including direct contact, exposure to respiratory secretions, and the release of aerosolized infectious particles from skin lesions. The initial infection is extremely contagious, and the secondary attack rate can reach nearly 100% among susceptible individuals living in the same household. After exposure, the incubation period typically lasts between 10 and 21 days. The period during which an infected person can spread the virus begins 1 to 2 days before the characteristic rash develops and continues until all skin lesions have fully crusted [8,9]. Primary varicella infections typically present with a characteristic itchy vesicular rash, fever, and malaise. The rash progresses from macules to papules to vesicles and finally to crusts. After initial infection, the virus is neurotropic, establishes latency in the dorsal root ganglia, and can be reactivated later in life, causing HZ. Its pathogenesis involves viral entry, replication, and spread, followed by an immune response that controls infection but does not eliminate the virus [8,10,11].

Following latency, VZV reactivation results in HZ, which is characterized by a painful, localized rash that follows a dermatomal distribution. The pain associated with HZ, called PHN, can persist for months to years and significantly affects quality of life, especially in older adults. Immunosuppression, aging, and stress increase the likelihood of VZV reactivation [12].

## Varicella vaccination

Immunization is recognized as the most effective approach for preventing diseases caused by VZV. The first varicella vaccine was developed in Japan in 1974 by Takahashi et al. [13] who produced the Oka-V strain by propagating the wild-type VZV strain (Oka-P) in human diploid cell cultures.

Since 1995, Merck (Merck & Co., Inc.; Whitehouse Station, NJ, USA) has marketed this strain as Varivax for use in healthy children aged ≥12 months. Furthermore, the combination vaccine ProQuad (Merck & Co., Inc.), which protects against measles, mumps, rubella, and varicella, has been licensed in the United States since 2005 by Merck (Merck & Co., Inc.) for healthy children between 12 months and 12 years of age [14].

In Korea, the varicella vaccine was incorporated into the National Immunization Program (NIP) in 2005, mandating a single dose for all children aged 12 to 15 months. This policy led to a rapid increase in vaccine uptake, with coverage rates exceeding 97% by 2011 [15]. Interestingly, despite this high coverage, the number of reported varicella cases paradoxically increased from 22.5 per 100,000 in 2006 to 154.8 per 100,000 in 2017 [16]. This trend has been attributed to several factors, including improved surveillance sensitivity, waning immunity associated with a single-dose schedule, and primary vaccine failure [17]. However, more granular analyses revealed a positive impact: incidence rates among specific birth cohorts vaccinated under the NIP have significantly decreased compared with pre-NIP cohorts, and the severity of breakthrough cases has been markedly attenuated. The debate continues regarding the potential benefit of transitioning to a two-dose schedule to further control breakthrough outbreaks, mirroring the strategies adopted in the United States [18].

Varicella vaccines have demonstrated remarkable efficacy in preventing initial infection in children, whereas shingles vaccines have contributed to a reduction in the incidence of complications from reactivation, particularly among older and immunocompromised individuals. Recent vaccine research has explored different approaches to increase immunity and provide sustained protection, which will play an important role in strengthening long-term prevention strategies against VZV infections [19].

The vaccine stimulates the immune system to produce antibodies against VZV, thereby providing immunity against the disease. It contains a weakened form of the virus that can stimulate the immune system without causing full-blown disease. By exposing the body to a weakened virus, the vaccine encourages the immune system to recognize and respond to future exposure to the varicella virus. The vaccine is highly effective in reducing the incidence of chickenpox as well as the disease severity in vaccinated individuals [10,20]. In countries with high vaccination coverage, the incidence of varicella has dramatically decreased, leading to a reduction in varicella-associated complications, such as pneumonia, encephalitis, and hospitalizations [21].

In the United States, the introduction of the vaccine in 1995 resulted in a 90% reduction in varicella-related hospitalizations and a 71% decrease in deaths from the disease [22]. Other studies have indicated that a single dose of the varicella vaccine is approximately 70% to 90% effective in preventing varicella infection and over 95% effective in preventing severe cases (Table 2) [23]. Varicella vaccinations not only directly protect individuals but also indirectly benefit the community by reducing virus circulation, leading to herd immunity effects [24].

However, owing to reports of breakthrough infections (i.e., milder cases in vaccinated individuals), a two-dose regimen was introduced, enhancing immunity and providing approximately 95% efficacy in preventing any form of varicella [25]. Research has confirmed that individuals who receive two doses exhibit stronger and longer-lasting immunity than those who receive a single dose [18].

The varicella vaccine has been shown to be safe, with most adverse effects being mild and self-limiting. Common adverse effects include redness and swelling at the injection site as well as a mild rash. In some cases, a systemic reaction may occur, manifesting as a low-grade fever, rash, or fatigue. These symptoms are generally mild and subside on their own within a few days. However, severe adverse effects have been reported in rare instances. These symptoms may include febrile convulsions or thrombocytopenia, and individuals with compromised immune systems are more susceptible to severe manifestations. Serious allergic reactions including anaphylaxis have also been reported. Adverse reactions to vaccinations are potential complications that require immediate medical attention [26].

A few studies have documented rare instances of post-varicella vaccination HZ. One study documented cases of HZ that occurred after varicella vaccination in healthy young children, and vaccine-induced HZ was identified in one of these cases [27]. A separate study documented a frequency of 16 cases per 100,000 individuals following varicella vaccination, which is lower than that observed from natural infection [28]. HZ has been observed to be associated with varicella vaccination, albeit with symptoms of a more moderate nature, compared to cases of varicella in individuals who are unvaccinated and develop HZ.

## Breakthrough varicella infections

Breakthrough varicella infections occur in individuals who develop varicella >42 days after vaccination. These cases are generally milder than those observed in unvaccinated individuals and present distinct clinical and epidemiological challenges. Subsequent episodes of HZ caused by the vaccine strain are also less severe and may carry a lower risk of PHN than HZ resulting from wild-type VZV [29,30].

Breakthrough infections have been observed in both single-dose and two-dose vaccine recipients, although they occurred more frequently in the former group. Recent estimates suggest that the cumulative incidence of breakthrough varicella among vaccinated individuals increases over time after vaccination, highlighting the need for long-term monitoring of vaccine-induced immunity [31]. Clinically, breakthrough varicella is characterized by the absence of vesicular lesions, with the rash presenting predominantly as macules or papules rather than vesicles. This eruption may resemble other common dermatological conditions, which can complicate an accurate clinical diagnosis. Fever and malaise are rare complications that generally resolve more rapidly than primary varicella [32].

Diagnosis is challenging because of the mild, atypical presentation and overlap with other dermatological conditions. Laboratory testing, such as polymerase chain reaction analysis of skin lesions, is the most reliable method for confirmation because clinical diagnosis alone is often insufficient [33].

Since the introduction of the varicella vaccine, the proportion of breakthrough varicella among all varicella cases has increased, largely due to a significant decline in the overall disease incidence. Despite this relative increase, the overall disease burden remains much lower than that in the pre-vaccine era because breakthrough infections are generally milder.

Breakthrough varicella is expected in populations with high vaccination coverage. Despite these breakthrough cases, the varicella vaccine has been tremendously successful in reducing the overall burden of disease, with effectiveness reaching 97% after two doses. Evidence suggests that primary vaccine failure, rather than waning immunity, accounts for most breakthrough cases, supporting current two-dose vaccination strategies.

The clinical profile of breakthrough varicella, with fewer lesions, milder symptoms, and a shorter duration, represents a significant benefit of vaccination, even when it does not completely prevent infection. As varicella vaccination programs continue to evolve, ongoing surveillance of breakthrough infections will remain important for optimizing vaccination strategies and maintaining the substantial public health benefits achieved to date.

Breakthrough varicella presents unique challenges in the post-vaccination era. Although these infections are less severe, their atypical presentation complicates their diagnosis and management. Ongoing research and surveillance are essential for optimizing vaccination strategies and minimizing the impact of breakthrough infections on public health.

## Impact of varicella vaccination on herpes zoster incidence

Prior to the introduction of universal varicella vaccination, mathematical models based on the “exogenous boosting” hypothesis predicted a substantial, temporary increase in HZ incidence among adults, owing to reduced exposure to wild-type VZV [34]. However, global epidemiological data from the past 20 years have largely contradicted these predictions.

In the United States, which implemented universal varicella vaccination in 1995, longitudinal studies have shown that while HZ incidence has been increasing, this upward trend began before the vaccination program started and did not accelerate after its introduction [6,35]. Leung et al. [6] and Hales et al. [35] demonstrated that age-specific HZ incidence rates continued to rise at a similar rate in the post-vaccination era as in the pre-vaccination era, suggesting that factors other than reduced boosting, such as aging populations, increased awareness, and diagnostic coding changes, are the primary drivers of this trend.

A systematic review by Kawai et al. [36] found that the incidence of HZ has been rising in many countries, regardless of whether routine varicella vaccinations were implemented. For instance, increasing HZ trends have been observed in countries without universal varicella vaccination programs, further challenging the notion that vaccination is the sole cause of rising HZ rates in adults.

**Fig. 1.**
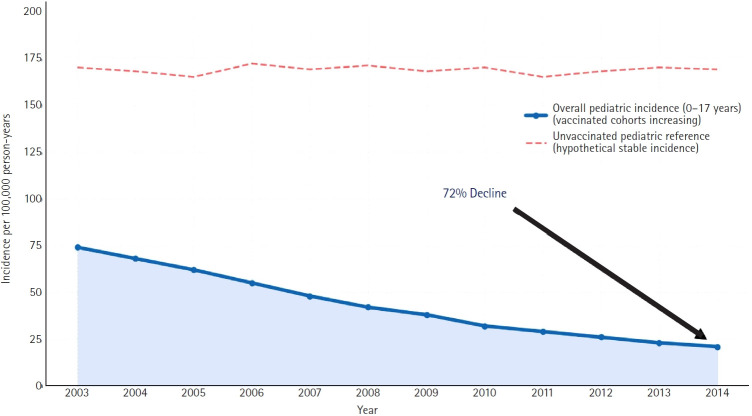
Trends in herpes zoster incidence among children in the United States (2003–2014). The solid blue line represents the overall annual incidence of herpes zoster per 100,000 person-years among children aged ≤17 years. Following the widespread implementation of the universal varicella vaccination program, the incidence of herpes zoster in the pediatric population declined by approximately 72% over the study period. This decline highlights the protective effect of the vaccine against herpes zoster in vaccinated birth cohorts, contrasting with the higher rates observed in unvaccinated historical cohorts. Adapted from Weinmann et al. [37] with permission from the American Academy of Pediatrics.

**Fig. 2.**
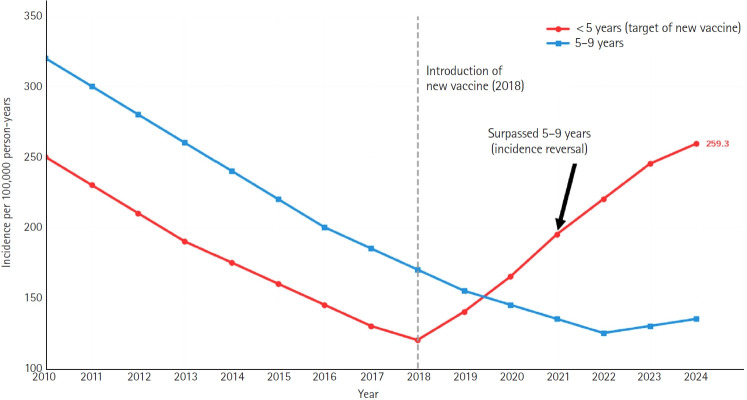
Annual incidence of herpes zoster per 100,000 person-years in children aged <5 years (red line) and 5 to 9 years (blue line). While the incidence in both age groups demonstrates a steady downward trend from 2010 to 2018, a significant trend reversal is observed in children aged <5 years starting in 2019, following the introduction of a new live-attenuated varicella vaccine into the National Immunization Program in 2018. The incidence rate in the <5-year group surpasses that of the 5- to 9-year group in 2021 and reached a record high of 259.3 cases per 100,000 person-years by 2024. This highlights an ongoing, large-scale herpes zoster outbreak associated with the recent vaccine introduction in the pediatric population. Adapted from Lee [38] with permission from Taylor & Francis Informa UK, Ltd.

Conversely, the impact on the vaccinated pediatric population is clearly beneficial. In regions with high vaccine coverage, such as the United States, the incidence of HZ among children has declined significantly, by >70% in some cohorts, demonstrating that the attenuated vaccine strain is less likely to reactivate than the wild-type virus (Fig. 1) [37]. The recent epidemiological shift observed in the Korean pediatric population introduces a critical nuance that transcends the traditional dichotomous debate on whether varicella vaccination simply “prevents” HZ or exacerbates adult cases through “reduced exogenous boosting.” The unprecedented surge in pediatric HZ cases following the introduction of a specific live-attenuated vaccine in 2018 highlights a novel paradigm: the profound impact of the inherent virological characteristics of the vaccine strain itself (Fig. 2) [38]. These data suggest that the long-term effects of a vaccination program on HZ epidemiology are strongly contingent on the latency and reactivation potential of the specific attenuated strain utilized. Consequently, a vaccine strain with higher reactogenicity can paradoxically trigger an explosive, large-scale outbreak of vaccine-derived HZ among recently immunized cohorts rather than functioning solely as a preventive measure. This striking phenomenon underscores the pressing need to move beyond evaluating vaccines based purely on their efficacy against primary varicella infections. Future public health policies and vaccine development must rigorously prioritize the long-term genomic stability and specific reactivation profiles of live-attenuated strains [38].

Modeling studies have also explored the potential impact of varicella vaccination on the incidence of HZ. One study used an age-structured dynamic transmission model to predict the impact of varicella vaccination on the incidence of HZ. The study found that, assuming modest levels of endogenous boosting, the increase in HZ incidence following childhood varicella vaccination was smaller and lasted for a shorter period compared to scenarios without endogenous boosting. These results suggest that the overall impact on HZ incidence was less significant than initially predicted [39].

## Temporal trends in herpes zoster incidence

To accurately assess the impact of varicella vaccination on HZ epidemiology, it is essential to examine long-term population-based data rather than rely solely on mathematical modeling. A critical synthesis of real-world evidence from various countries has revealed a consistent upward trend in HZ incidence that appears to be largely independent of childhood varicella vaccination programs.

In the United States, where universal varicella vaccination began in 1995, data from the Centers for Disease Control and Prevention and managed care organizations have provided the most extensive evidence. A pivotal study by Hales et al. [35], which analyzed Medicare data from 1993 to 2010, found that HZ incidence increased by 39% over the study period. However, the rate of increase did not accelerate after the introduction of the vaccination program. Furthermore, the HZ incidence was found to be increasing, even in states with low vaccine coverage, contradicting the hypothesis that reduced exogenous boosting from vaccinated children drives adult HZ rates [6,35].

Evidence from countries without universal varicella vaccination further challenges the link between vaccination and increasing HZ rates. In the United Kingdom and many European nations, where routine varicella vaccination was not implemented during the study period, the HZ incidence has predominantly shown an upward trajectory [40]. A systematic review by Kawai et al. [36] confirmed that HZ incidence is increasing globally, regardless of varicella vaccination policies. This suggests that the rising burden of HZ is driven by other factors, such as population aging, immunosenescence, and arguably increased medical attention or diagnostic coding practices, rather than by the absence of wild-type VZV circulation.

Data from Korea offer a unique perspective. Following the inclusion of the varicella vaccine in the NIP in 2005, coverage rates exceeded 97%. The incidence of HZ demonstrated a steady upward trend beginning in 2010, reaching a peak in 2018, followed by a period of stabilization, and a slight decline through 2022. In 2010, the incidence rate was 8.1 cases per 1,000 person-years. This rate increased consistently over subsequent years, culminating in a peak of 11.9 cases per 1,000 person-years in 2018. After 2018, the incidence showed a modest decrease, declining to 10.7 cases per 1,000 person-years by 2022, suggesting stabilization, with a slight downward trend in recent years [41]. This decline may have been influenced by several factors, such as adherence to hygiene guidelines during the coronavirus disease 2019 pandemic and the expansion of vaccination programs.

## Herpes zoster and postherpetic neuralgia after varicella vaccination

The varicella vaccine offers protection against primary VZV infections. This vaccine has been shown to reduce viral load in the body and the likelihood of viral reactivation into HZ in adulthood. Research has demonstrated that individuals who receive the varicella vaccine during childhood exhibit a reduced incidence of HZ compared with those who experience a natural varicella infection. In the event of vaccination and the subsequent development of HZ, the manifestations are typically less severe, characterized by a reduced number of skin lesions and diminished intensity of pain. PHN has been found to be strongly associated with severe HZ outbreaks; therefore, if a patient experiences milder symptoms, the likelihood of developing PHN is also reduced [42,43].

The efficacy of varicella vaccination in mitigating the risk of PHN has been well documented; however, this vaccine does not eliminate the risk of PHN. Although some recipients may still develop HZ, the risk of progression to PHN is mitigated when cases are less frequent, less severe, and milder. Therefore, in adults, particularly those aged ≥50 years, the administration of an HZ vaccine provides additional protection against both HZ and PHN. The vaccine has been found to be highly effective and is recommended for individuals who received varicella vaccination during childhood [30].

The reduction in circulating wild-type VZV due to vaccination has led to concerns about decreased natural immune boosting in older adults, potentially increasing the risk of HZ and PHN [44]. Nevertheless, studies have suggested that the introduction of the HZ vaccine in older adults has effectively mitigated this risk, reducing both HZ and PHN incidences in this demographic [19].

The varicella vaccine has significantly impacted the epidemiology of varicella and HZ. While the incidence of PHN remains a concern, ongoing research and surveillance are essential to fully understand the long-term effects of varicella vaccination on the incidence of PHN and to optimize vaccination strategies. With the widespread implementation of varicella vaccination programs, the epidemiology of HZ and PHN is undergoing significant changes, warranting the reassessment of preventive measures and management strategies [45].

Varicella vaccination activates the production of VZV-specific T cells, which may prevent viral reactivation and indirectly reduce the risk of HZ. We do not yet have sufficient data on the long-term efficacy of the varicella vaccine, because children who received it in their first year of life in 1995 will be 50 years old in 2044 [46]. Other data have shown different trends in different age groups because of vaccination programs, suggesting that national varicella vaccination and the introduction of the HZ vaccine may have influenced the incidence of HZ. As different trends are observed in different age groups, ongoing surveillance of HZ is needed to better understand these changes and inform the development of targeted prevention strategies. This study highlights the importance of monitoring the epidemiology of HZ in the context of changing demographics and vaccination policies [47].

## Herpes zoster vaccine for older individuals

The HZ vaccine was designed to reduce the incidence and severity of shingles, particularly in those who are older [48]. There are two main types of HZ vaccines: live-attenuated zoster vaccine (Zostavax; Merck & Co.) and recombinant zoster vaccine (Shingrix; GlaxoSmithKline, Brentford, UK). The live-attenuated zoster vaccine was the first to be approved for use in adults aged ≥50 years [49], although the recombinant zoster vaccine has shown higher efficacy and is recommended for adults in this age group [48]. Studies have demonstrated that the recombinant zoster vaccine is more effective than the live-attenuated zoster vaccine in preventing HZ and PHN. A systematic review and network meta-analysis found that the recombinant zoster vaccine demonstrated 85% efficacy and was associated with fewer serious adverse events than the live-attenuated zoster vaccine [50].

Both vaccines are generally well tolerated; however, the recombinant zoster vaccine is associated with more frequent and severe injection-site reactions. Despite this, the overall safety profile of the recombinant zoster vaccine is favorable and is not contraindicated in individuals who are immunocompromised. Adults who are immunocompromised are at an increased risk of developing HZ and its associated complications. The recombinant zoster vaccine is approved for use in adults aged ≥19 years who are immunocompromised. The recombinant zoster vaccine has been shown to be effective in this population, with an efficacy of 60% [49].

## Future directions and considerations of varicella vaccination

Varicella vaccination programs have significantly reduced the incidence and severity of varicella globally, demonstrating their high efficacy in preventing disease and associated complications. Despite these successes, challenges remain, including the management of HZ risks, breakthrough varicella infections, and optimization of vaccination schedules. As new data emerge, future strategies need to address these challenges while integrating novel vaccine technologies, improving coverage, and adapting to evolving epidemiological trends.

Continued efforts to increase vaccination coverage, particularly among the underserved populations, are crucial. Innovative strategies such as school-based vaccination programs and community outreach can help achieve higher immunization rates. Research into new vaccine formulations, including those that provide long-lasting immunity or require fewer doses, is ongoing. These advancements could improve vaccine uptake and adherence. Expanding vaccination programs in low- and middle-income countries is essential to reduce the global burden of varicella and prevent outbreaks. International collaboration and funding will be key to these efforts.

The ongoing monitoring of vaccine safety and efficacy is essential. Surveillance systems should be strengthened to detect and respond to adverse events or changes in vaccine effectiveness. Achieving and maintaining herd immunity is critical for protecting individuals who cannot be vaccinated, such as those who are immunocompromised. Public health campaigns should emphasize the importance of vaccination for community protection. Regular updates on vaccination policies and recommendations based on the latest scientific evidence are necessary. This includes revising age-specific vaccination schedules and catch-up programs for older children and adults. Clear and accurate communication regarding the benefits and risks of varicella vaccinations is vital to address vaccine hesitancy.

## Conclusion

Varicella vaccination programs have significantly reduced the incidence of varicella, thereby positively contributing to public health. However, their effects on HZ outbreaks and PHN remain complex and require ongoing monitoring and further research. The introduction of the HZ vaccine has provided an additional layer of protection, addressing the needs of older adults who are at higher risk for HZ and PHN.

Further research is needed to fully understand the long-term impact of varicella vaccination on the incidence of HZ and PHN and to identify additional risk factors for them. Ongoing studies will help to refine vaccination strategies and improve the management of HZ and PHN. Advancements in vaccine formulations such as adjuvanted vaccines are expected to enhance immunity against both varicella and HZ, potentially eliminating the risk of PHN.

## Figures and Tables

**Table 1.** Classification of human herpesvirus

Virus	Number (HHV)	Type	Transmission route	Associated disease and key features	Diagnostic methods	Treatment
Herpes simplex virus 1	HHV-1	Alpha	Saliva, mucosal contact, skin-to-skin	Causes cold sores, primarily affects oral and facial areas	Clinical symptoms, PCR, viral culture	Antivirals (e.g., acyclovir, valacyclovir)
Herpes simplex virus 2	HHV-2	Alpha	Sexual contact, vertical transmission (birth)	Genital herpes, neonatal infections; transmitted via sexual contact	Clinical symptoms, PCR, serological tests	Antivirals (e.g., acyclovir, valacyclovir)
Varicella zoster virus	HHV-3	Alpha	Respiratory droplets, skin lesion contact	Causes chickenpox and shingles; reactivates later as shingles	Clinical symptoms, serological tests, PCR	Antivirals (e.g., acyclovir), vaccines
Epstein-Barr virus	HHV-4	Gamma	Saliva, body fluid contact	Infects B cells; mononucleosis, Burkitt lymphoma, and nasopharyngeal carcinoma	Serological tests, PCR, atypical lymphocytes	Supportive care, antivirals in immunocompromised cases
Cytomegalovirus	HHV-5	Beta	Bodily fluids, vertical transmission, transplants	Mostly asymptomatic but severe in immunocompromised individuals (e.g., pneumonia, retinitis)	PCR, serological tests, tissue biopsy	Antivirals (e.g., ganciclovir, foscarnet)
Human herpesvirus 6	HHV-6	Beta	Saliva, respiratory droplets	Causes roseola in infants; can lead to encephalitis in immunocompromised adults	Clinical symptoms, PCR, serological tests	Supportive care, antivirals in severe cases
Human herpesvirus 7	HHV-7	Beta	Saliva, respiratory droplets	Similar to HHV-6; also associated with roseola	PCR, clinical symptoms	Supportive care
Kaposi sarcoma-associated herpesvirus	HHV-8	Gamma	Saliva, bodily fluids, transplants	Associated with Kaposi sarcoma and lymphoproliferative disorders in immunocompromised patients	Tissue biopsy, immunohistochemistry, PCR	Antivirals, cancer therapy (e.g., for Kaposi’s sarcoma)

HHV, human herpesvirus; PCR, polymerase chain reaction.

**Table 2.** Reduction in varicella incidence after vaccine introduction by countries

Country	Year of vaccine introduction	Study period	Pre-vaccine incidence (per 100,000)	Post-vaccine incidence (per 100,000)	Reduction rate (%)
USA	1995	1995–2005	1,600	300	81
Germany	2004	2004–2014	1,400	200	86
South Korea	2005	2005–2015	1,500	250	83
Japan	2014	2014–2021	1,300	150	88

Source: Centers for Disease Control and Prevention, Robert Koch Institute, Korea Disease Control and Prevention Agency, and Japan Ministry of Health, Labour and Welfare.
